# Ribosomal RNA-derived fragments in cancer: molecular identity, cellular context, and functional evidence

**DOI:** 10.3389/fcell.2026.1950076

**Published:** 2026-10-22

**Authors:** Xianfang Shao, Han Li, Meiying Mu, Zhenzhen Ma

**Affiliations:** 1 Department of Critical Care Medicine, Zibo Central Hospital, Zibo, Shandong, China; 2 Department of Oncology, Zibo Central Hospital, Zibo, Shandong, China

**Keywords:** cancer, extracellular RNA, liquid biopsy, post-transcriptional regulation, ribosomal RNA-derived fragments, RNA modification, rRNA processing, subcellular localization

## Abstract

Ribosomal RNA-derived fragments (rRFs), also termed rRNA-derived small RNAs (rsRNAs), occur in cells and biofluids, but their detection does not establish biological activity. This review systematically summarizes current research on rRF biogenesis, intracellular functions, extracellular localization and carriage, and clinical associations, while emphasizing that the detection of rRFs does not in itself establish biological function. Stress-associated cleavage, candidate-specific non-canonical processing, and extracellular ribosome fragmentation can each contribute to the observed repertoire. Functional cancer studies remain concentrated in a few candidates: depletion of a 28S-derived rRF3 affects H1299 cell phenotypes; rsRNA-28S inhibition increases PTGIS expression and taxane resistance in prostate-cancer cells; and synthetic gastric-cancer S2 and S7 mimics alter target reporters and protein abundance. These findings do not establish a common rRF pathway or therapeutic efficacy. Tissue and circulating signatures show diagnostic or prognostic associations, including a methylation-sensitive 28S fragment assay, but many cohorts are small and lack prospective external validation. Therapeutic development could test inhibition of a confirmed tumor-promoting fragment or replacement of a protective fragment, provided that endogenous function, selectivity over parental rRNA, delivery, and safety are established. Overall, rRFs show emerging biological and biomarker potential in cancer, but their clinical translation will require stronger mechanistic validation, prospective clinical evidence, and rigorous assessment of delivery and safety.

## 1 Introduction

Fragments derived from abundant structural RNAs were historically regarded as degradation products or sequencing by-products (Ravi Kumar et al., 2025). Human rRNAs nevertheless generate reproducible fragment profiles that vary with parental locus, tissue, disease state, sex, and population background (Cherlin et al., 2020; Pliatsika et al., 2024). Some fragments occur in Argonaute-associated datasets and RNA interaction chimeras, supporting the possibility of sequence-specific regulation (Guan and Grigoriev, 2021; Guan et al., 2025). These findings suggest that rRFs warrant investigation as distinct molecular entities, but their reproducible detection should not be equated with demonstrated biological function.

Cancer studies address different questions. Tissue and blood profiling measure disease-associated abundance or chemistry, whereas perturbation experiments test candidate activity (Yang et al., 2025; Gu et al., 2025; Pu et al., 2025; Zhan et al., 2025; Sanchez-Delgado et al., 2025; Gu et al., 2020; Diamantop et al., 2024; Chen et al., 2017; Qiao et al., 2023). Extracellular-RNA studies add information about carriers and processing outside cells (Tosar et al., 2020; Murillo et al., 2019; Srinivasan et al., 2019; Chen et al., 2024). These findings should not be combined into a single causal model. A diagnostic rRF signal may not originate from viable tumor cells, while an intracellular regulatory rRF may not necessarily be detectable in the circulation.

We first describe biogenesis and classification, then intracellular mechanisms and extracellular context, and finally biomarker applications, therapeutic opportunities, and limitations. Table 1 summarizes the principal cancer studies; Figures 1–3 illustrate fragment origins, biogenesis routes, and target regulation.

**TABLE 1:** Evidence from major cancer-associated rRF studies.

Study and model	Material and principal result	Evidence supported	Main limitation
Chen et al. (2017) H1299 cells	20-nt 28S rRF3 depletion increased apoptosis and reduced proliferation.	Candidate-associated cellular phenotype.	Target unresolved; vector-based depletion needs fragment-specific rescue and parental-rRNA controls.
Qiao et al. (2023) Prostate cancer	DU-145/DU-145R: rsRNA-28S ASO increased PTGIS and taxane resistance; mutant reporter lost the response.	Site-dependent target regulation and an *in vitro* treatment phenotype.	No therapeutic replacement or *in vivo* rescue. Patient comparison: n = 23, chemotherapy-associated reduction not significant (P = 0.352).
Yang et al. (2025) Gastric cancer	Mixed S2/S7 rRF plus S10 tRF plasma signature: AUC 0.91 in 143 cases/167 controls; test-cohort AUCs 0.97 and 0.91.	Diagnostic association and mimic-induced reporter/protein responses.	EV-enriched material does not prove intravesicular tumor RNA; endogenous S2/S7 necessity and therapeutic benefit untested.
Gu et al. (2025) Colorectal cancer	Six plasma rsRNAs: AUC 0.898; with conventional markers, 0.942.	Exploratory plasma diagnostic panel.	Carrier and mechanism unresolved; reported discrimination does not establish prospective clinical utility.
Pu et al. (2025) Hypopharyngeal cancer	Five paired tissues: altered rsRNA fraction, length, and parental composition.	Disease-associated repertoire under PANDORA-seq conditions.	Small descriptive cohort; relative fractions do not measure absolute abundance or mechanism.
Zhan et al. (2025) Five cancer types	120 serum samples; six-exRNA classifier included five rsRNAs.	Internal proof of principle for cancer classification.	Held-out samples derive from the same cohort; case/control counts are inconsistent in parts of the source Results.
Sanchez-Delgado et al. (2025) Suspected lung cancer	28S4014–4035 methylation states in 27 cases and 16 non-cancer controls; AUC 0.84.	Fragment chemistry can carry diagnostic information.	Small, selected cohort; external validity, tumor source, and biological function unresolved.
Gu et al. (2020) Lung cancer	PBMC TRY-RNA signature includes tRNA-, rRNA-, and YRNA-derived species.	Blood-cell-associated mixed RNA signature.	Neither a cell-free assay nor an rRF-only effect; host-cell composition may contribute.
Diamantopoulos et al. (2024) Prostate tissue	89 cancers/53 benign samples: lower overall cancer levels; higher within-cancer levels associated with relapse.	Tissue diagnostic and internally validated prognostic association.	Observational; no causal mechanism; identity relative to the PTGIS-associated fragment unconfirmed.

AUC, area under the receiver-operating characteristic curve; ASO, antisense oligonucleotide; EV, extracellular vesicle; PBMC, peripheral blood mononuclear cell. Reported AUCs reflect different populations and tasks and are not directly comparable.

**FIGURE 1:**
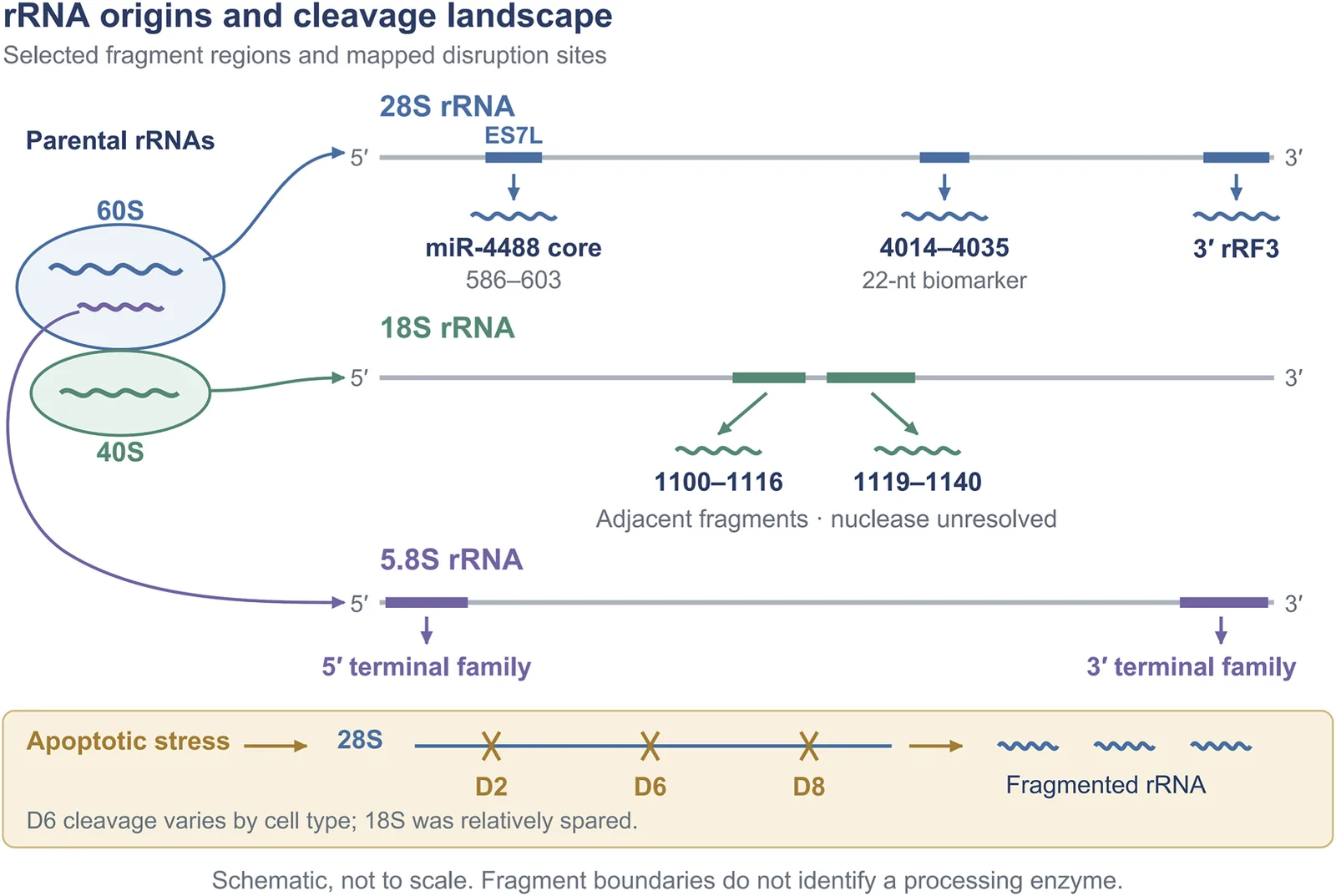
rRNA origins and cleavage landscape. The maps are schematic and not to scale. Colored segments indicate reported fragment regions or terminal families. Examples include the miR-4488 core at 28S ES7L 586–603, the circulating 28S 4014–4035 fragment, terminal 28S and 5.8S rRF families, and adjacent 18S fragments 1100–1116 and 1119–1140 (Guan and Grigoriev, 2021; Sanchez-Delgado et al., 2025; Chen et al., 2017; Michael et al., 2026). Ochre markers indicate the D2, D6, and D8 cleavage regions identified in apoptosis-related studies (Houge et al., 1995). Coordinates follow the reference conventions used in the original studies. The ribosome schematic indicates parental rRNA origin, while connector arrows denote fragment origin rather than a single cleavage pathway.

**FIGURE 2:**
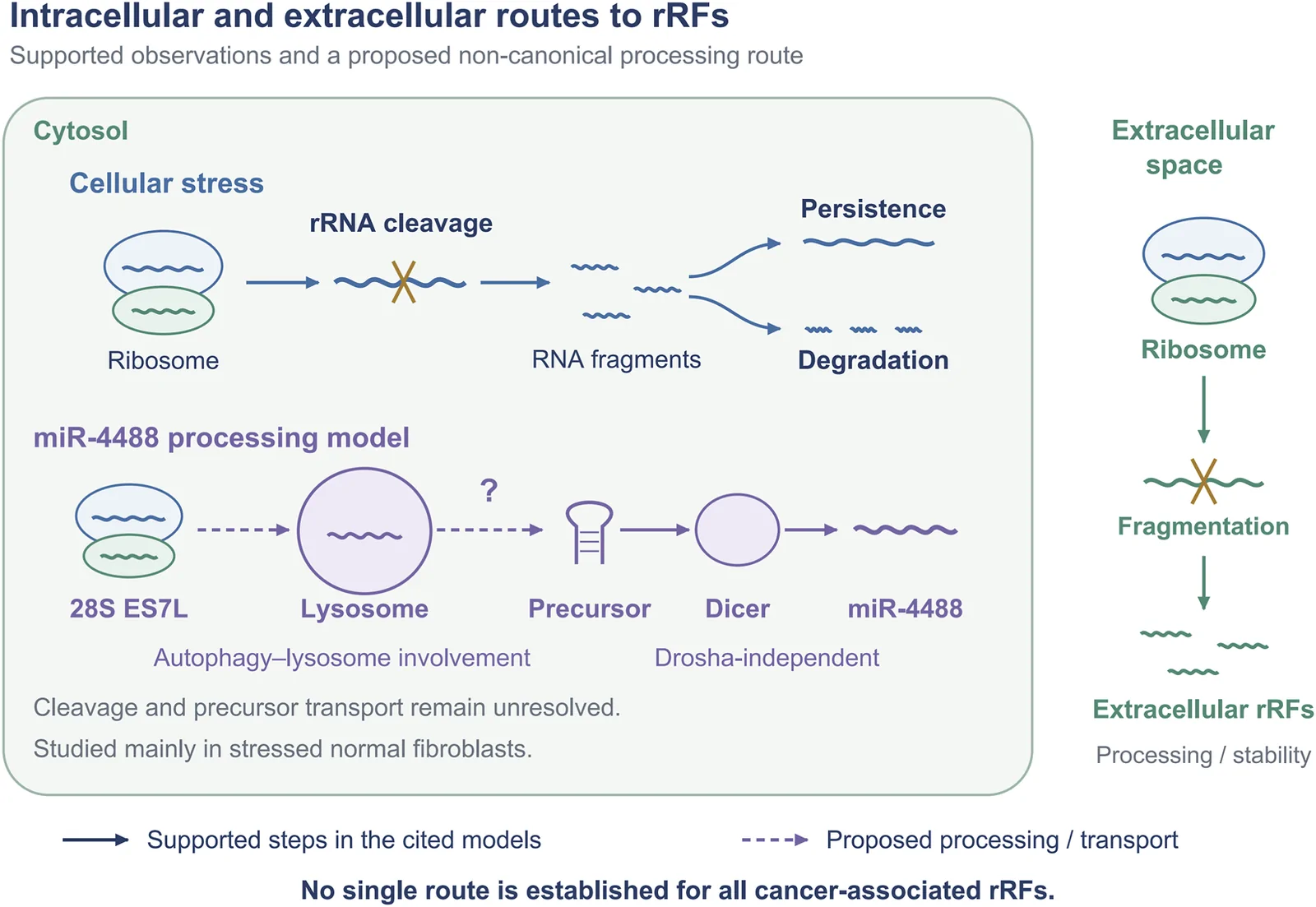
Intracellular and extracellular pathways contributing to rRF biogenesis. Stress-associated rRNA disruption, autophagy–lysosome-dependent processing of miR-4488, and extracellular ribosome fragmentation represent distinct routes that may contribute to the rRF repertoire (Tosar et al., 2020; Houge et al., 1995; Michael et al., 2026; Butler et al., 2023). In stressed fibroblasts, miR-4488 production was Dicer-dependent, Drosha-independent, and required the autophagy–lysosome pathway (Michael et al., 2026). Dashed arrows indicate proposed processing or transport steps, and the question mark denotes an unresolved transport step. The schematic summarizes these context-dependent pathways without implying that they occur simultaneously in the same cell.

**FIGURE 3:**
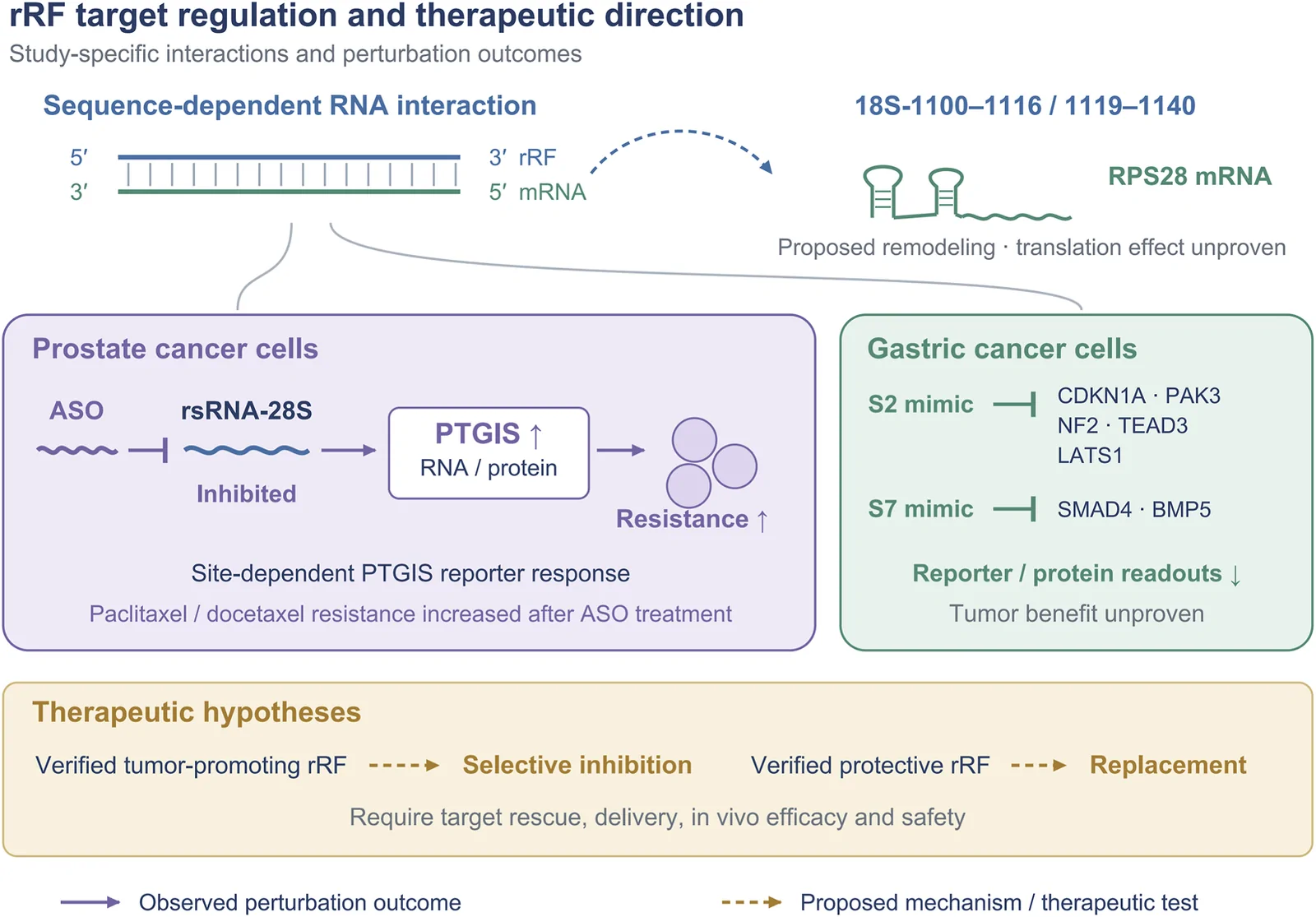
rRF target regulation and therapeutic directions. The schematic summarizes representative mechanisms of rRF-mediated regulation, including the proposed interaction of adjacent 18S-derived fragments with RPS28 mRNA, rsRNA-28S–PTGIS regulation in prostate-cancer cells, and S2/S7-mediated target regulation in gastric-cancer cells (Guan and Grigoriev, 2021; Guan et al., 2025; Yang et al., 2025; Qiao et al., 2023). In DU-145/DU-145R cells, inhibition of rsRNA-28S increased PTGIS expression and taxane resistance, while S2/S7 mimics altered target-reporter activity and corresponding protein expression. Potential therapeutic approaches include inhibition of tumor-promoting rRFs and replacement of protective rRFs. Solid arrows indicate experimentally observed effects, whereas dashed arrows denote proposed mechanisms or therapeutic strategies. ASO, antisense oligonucleotide; PTGIS, prostaglandin I2 synthase.

### 1.1 Review scope and literature identification

This structured narrative review considered PubMed-indexed literature available through 25 July 2026. It was not designed as a systematic review or meta-analysis. PubMed was the database used for literature identification. The search terms included “ribosomal RNA-derived fragment,” “rRF,” “rsRNA,” “rRNA-derived small RNA,” “cancer,” “tumor,” “extracellular RNA,” “extracellular vesicle,” “PANDORA-seq,” “RNA modification,” “Argonaute,” “translation,” and “innate immune sensing.” Combinations of these terms were used to identify literature related to rRF identity, cancer, analytical methods, and biological context. Eligible cancer studies evaluated rRF abundance or chemistry, cellular phenotypes, target regulation, treatment response, or clinical performance. Non-cancer studies were included when they provided relevant mechanistic context for rRF processing, localization, carriers, or receptor interactions. Studies focused exclusively on tRNA-derived fragments were not considered direct evidence for rRFs. As this was a narrative review rather than a systematic review or meta-analysis, no formal study-selection flow or quantitative assessment of publication bias was performed.

## 2 Biogenesis and classification of rRFs

### 2.1 Parental sources and fragment classes

Nuclear 18S, 5.8S, and 28S rRNAs arise from a common pre-rRNA through nucleolar processing, folding, chemical modification, and ribosomal-protein assembly; 5S rRNA is transcribed separately (Cherlin et al., 2020; Watkins and Bohnsack, 2012; Sloan et al., 2017). Fragments can also originate from mitochondrial 12S and 16S rRNAs or from pre-rRNA spacer regions (Pliatsika et al., 2024; Shi et al., 2018). These sources should be annotated separately because they differ in processing and compartmental context.

Based on their positions within the parental rRNA, rRFs can be broadly classified as 5′-derived, 3′-derived, or internal fragments. The terms rRF5 and rRF3 have been used to describe groups of similarly sized fragments originating from the termini of rRNAs, particularly from 5.8S and 28S rRNAs (Chen et al., 2017). In addition to these terminal species, fragments derived from internal rRNA regions and from pre-rRNA processing intermediates also contribute to the diversity of the rRF repertoire (Cherlin et al., 2020; Pliatsika et al., 2024; Guan and Grigoriev, 2021). Importantly, this position-based nomenclature primarily reflects fragment origin and does not, by itself, define the responsible nuclease, biogenesis pathway, or regulatory function.

The defining molecular unit should include the parental rRNA, reference sequence, genomic or transcript coordinate, strand, exact 5′ and 3′ ends, length, and, when relevant, terminal or internal chemistry (Pliatsika et al., 2024; Guan et al., 2025; Shi et al., 2018). Recurrent end positions strengthen the argument for non-random processing, but recurrence alone does not identify the responsible nuclease or establish biological activity (Cherlin et al., 2020; Guan and Grigoriev, 2021; Chen et al., 2017). Potential ambiguity among repeated rDNA loci and homologous nuclear, mitochondrial, or microbial rRNA sequences should be reported explicitly instead of being folded into a generic “rRNA-derived” category (Pliatsika et al., 2024; Shi et al., 2018; Ju et al., 2026).

SPORTS1.0 and MINRbase provide useful resources for annotating the parental origin and sequence identity of rRFs (Pliatsika et al., 2024; Shi et al., 2018). However, rRF nomenclature remains inconsistent across studies. Labels such as “rsRNA-28S” often indicate only the parental rRNA and do not uniquely define a specific molecular species. Because closely related rRF isoforms may differ by only a few nucleotides yet show distinct subcellular distributions and interaction profiles (Guan et al., 2025; Cherlin et al., 2024), precise sequence-level definition is important for cross-study comparison and functional interpretation.

### 2.2 Cleavage regions and non-canonical processing

Experimentally mapped cleavage regions and observed fragment boundaries represent distinct types of evidence, as summarized in Figure 1. Apoptosis-associated cuts were mapped to the D2 and D8 domains of 28S rRNA, with D6 cleavage reported in endothelial cells; 18S rRNA was relatively spared in those experiments (Houge et al., 1995). These large-rRNA disruption products are not automatically stable regulatory small RNAs. In contrast, profiling studies identified 5′- and 3′-terminal rRF families from 5.8S and 28S rRNAs, with no equivalent terminal enrichment for 18S in the study by Chen et al. (Chen et al., 2017). The different patterns argue against one universal cleavage program.

Selected sequence-resolved examples further illustrate this diversity. Human Ago-associated analyses identified adjacent 18S fragments designated 18S-1100–1116 and 18S-1119–1140 (Guan and Grigoriev, 2021). The miR-4488 core sequence maps to nucleotides 586–603 within the 28S ES7L region in the reference used by Michael et al. (Michael et al., 2026), whereas the circulating 28S4014–4035 fragment represents a distinct 22-nucleotide species (Sanchez-Delgado et al., 2025). Coordinates in these studies are reference-dependent. Observing a fragment with defined ends does not by itself identify the cleavage enzyme or prove that each end was generated in one step.

Cellular stress can produce structured rRNA breakdown. Chemotherapy, oxidative or endoplasmic-reticulum stress, and nutrient limitation have been associated with RNA disruption and loss of proliferative capacity (Butler et al., 2023). A candidate-specific non-canonical route has been described for miR-4488: in a study centered on metabolically stressed normal fibroblasts, its production was Drosha-independent, sensitive to impaired Dicer activity, and dependent on the autophagy–lysosome pathway (Michael et al., 2026). These findings support a lysosome-associated processing pathway for miR-4488, while the precise cleavage machinery and precursor trafficking remain incompletely understood.

Extracellular ribosomes can also undergo organized fragmentation, generating sequences after their release from cells (Tosar et al., 2020). These distinct intracellular and extracellular routes are summarized in Figure 2. Together, they suggest that rRF biogenesis may arise through multiple context-dependent pathways rather than a single conserved mechanism. Clarifying the origin of individual rRFs in cancer will require linking fragment dynamics to their parental rRNAs and candidate processing factors, while accounting for cell death and fragment stability.

### 2.3 Parental ribosome remodeling and modification

Cancer can alter the substrate from which rRFs arise. Marcel et al. showed that p53 represses fibrillarin expression and that loss of this control changes rRNA methylation, translational fidelity, and internal ribosome entry site-dependent translation (Marcel et al., 2013). Groza et al. subsequently linked fibrillarin depletion in triple-negative breast-cancer models to site-dependent 2′-O-methylation changes, altered 18S structure, reduced RPS28 incorporation, and changes in oncogenic translation (Groza et al., 2026). These findings highlight remodeling of the parental ribosome as an important context for rRF biology, while the contribution of individual rRFs to these phenotypes remains to be clarified.

Modification occupancy varies across rRNA sites and molecular populations (Sloan et al., 2017; Sharma et al., 2017). Dyskerin depletion can alter the translational properties of ribosomes (Penzo et al., 2015), while SNORA13 regulates ribosome biogenesis and senescence through an RPL23-associated route distinct from its modification-guide activity (Cheng et al., 2024). These mechanisms should be separated from fragment function. A parental modification can affect an rRF only if the relevant nucleotide is retained, or if it changes processing or stability.

### 2.4 Analytical detection and molecular identity

Conventional small-RNA libraries favor adapter-compatible termini and efficient reverse transcription, making sequence, structure, and chemistry determinants of detection (Jayaprakash et al., 2011). PANDORA-seq combines enzymatic treatments to recover additional RNA species that standard workflows capture poorly (Shi et al., 2021). The terminal-modification-independent cfRNA method described by Wang et al. reduces dependence on native 5′-end chemistry, rather than eliminating every source of library bias (Wang et al., 2024). Accordingly, these approaches improve the detection of RNA species that are poorly captured by conventional methods, thereby expanding the observable RNA repertoire.

End repair and demethylation treatments can substantially improve the sequencing visibility of certain rRFs, highlighting the influence of terminal chemistry and internal modifications on their detection. Beyond sequencing-based approaches, stable-isotope mass spectrometry can quantify rRNA modifications and provide complementary information on modification type and abundance (Popova and Williamson, 2014). More recently, targeted nanopore sequencing has enabled direct analysis of modification states in specific rRNA-derived fragments, offering a new approach for detailed characterization of rRF chemistry (Sanchez-Delgado et al., 2025).

## 3 Intracellular mechanisms and cancer phenotypes

### 3.1 Localization and access to effectors

Cherlin et al. used Fraction-seq in three triple-negative breast-cancer cell lines to examine nuclear, cytoplasmic, mitochondrial, and mitoplast fractions (Cherlin et al., 2024). After modeling cross-fraction contamination, they found sequence- and cell-type-dependent distributions, including differences between isoforms separated by only a few nucleotides. These findings highlight substantial heterogeneity in the subcellular distribution of rRFs and suggest that localization is likely to influence their access to molecular effectors and, consequently, their functional potential.

Argonaute-associated reads and guide–target chimeras suggest that a subset of rRFs may participate in sequence-specific RNA regulation (Guan and Grigoriev, 2021; Guan et al., 2025). Ribosome-associated signals further point to a close relationship between rRFs and their parental ribosomal context. Together, these observations indicate that rRF activity is likely shaped not only by sequence, but also by subcellular localization, molecular partners, and biochemical state. These factors are also important when interpreting perturbation experiments using synthetic mimics.

### 3.2 RNA duplexes and post-transcriptional regulation

RNA duplex formation offers a plausible route to target recognition. Ago-associated chimeras join an rRF sequence to an interacting RNA, while computational folding estimates the pairing pattern. These are different types of evidence: a chimera supports proximity or interaction under the capture conditions; a predicted duplex does not prove repression, cleavage, or altered translation. rRFtargetDB catalogs Ago1-associated candidates and predicted interaction properties rather than validating each pair in cancer (Guan and Grigoriev, 2021; Guan et al., 2025).

For example, the adjacent 18S-1100–1116 and 18S-1119–1140 fragments arise from a hairpin region and were associated with complementary regions of RPS28 mRNA in the computational analysis by Guan and Grigoriev (Guan and Grigoriev, 2021). This proposed mechanism provides a structural model for rRF–mRNA interaction and is summarized in Figure 3 together with experimentally characterized regulatory examples in cancer cells.

The prostate-cancer rsRNA-28S–PTGIS model includes a more direct sequence-specific test. Antisense inhibition of rsRNA-28S increased PTGIS expression and wild-type PTGIS 3′-UTR reporter activity in DU-145 and taxane-resistant DU-145R cells; mutation of the proposed site removed the reporter response (Qiao et al., 2023). These findings support site-dependent regulation of PTGIS by rsRNA-28S, with changes observed at both the RNA and protein levels.

In gastric-cancer cells, synthetic S2 and S7 mimics altered target reporters and reduced corresponding protein levels (Yang et al., 2025). Tested S2-associated targets included CDKN1A, PAK3, NF2, TEAD3, and LATS1; S7-associated targets included SMAD4 and BMP5. These findings show that S2 and S7 can modulate specific target reporters and protein expression in gastric-cancer cells, as summarized in Figure 3.

### 3.3 Cellular phenotypes and treatment response

Vector-based depletion of a 20-nucleotide 28S-derived rRF3 increased apoptosis, reduced proliferation, and altered cell-cycle distribution in H1299 cells (Chen et al., 2017). In prostate-cancer cells, rsRNA-28S inhibition increased viability during paclitaxel or docetaxel exposure and enhanced colony- and tumorsphere-associated phenotypes, while PTGIS overexpression similarly increased drug resistance (Qiao et al., 2023). In the clinical cohort of 23 patients, blood rsRNA-28S levels were lower in chemotherapy-treated patients, although the difference was not statistically significant (P = 0.352) (Qiao et al., 2023). Together, these findings suggest a potential role for specific rRFs in cancer-cell phenotypes and treatment response.

### 3.4 Translation and innate immune signaling

Translation-related effects provide another potential dimension of rRF biology. Studies of other small-RNA classes have shown that angiogenin-generated tRNA fragments can repress translation or inhibit translation initiation (Yamasaki et al., 2009; Ivanov et al., 2011), In parallel, perturbation of fibrillarin or dyskerin alters ribosome composition and translational activity (Marcel et al., 2013; Groza et al., 2026; Penzo et al., 2015). Highlighting the close relationship between rRNA processing, ribosome state, and translational control. Treatment-associated RNA disruption (Butler et al., 2023) and cellular stress accompanying immunogenic cell death (Fucikova et al., 2020) further illustrate how changes in RNA metabolism may emerge during cancer therapy.

Innate immune recognition of RNA is influenced by sequence, terminal chemistry, and RNA processing. Alharbi et al. showed that RNA fragments carrying a 5′-terminal 2′-O-methylguanosine can antagonize TLR7 and TLR8 in inflammatory models (Alharbi et al., 2026), while RNase T2- and RNase 2-dependent processing contributes to TLR8 recognition of RNA substrates (Ostendorf et al., 2020). Together, these findings provide a mechanistic basis for considering how the sequence and chemical features of rRFs may influence immune sensing in cancer.

## 4 Extracellular rRFs and intercellular mechanisms

### 4.1 Release and extracellular processing

Extracellular rRFs can reflect release from viable cells, cell damage, or processing of extracellular ribosomal material. Tosar et al. demonstrated that fragmentation of extracellular ribosomes contributes to the extracellular RNA repertoire (Tosar et al., 2020). These findings suggest that some extracellular rRFs may be generated or further processed after release from cells, highlighting extracellular processing as an additional source of rRF diversity.

### 4.2 Carriers and functional delivery

The exRNA Atlas describes RNA cargo associated with vesicles, lipoproteins, and ribonucleoprotein complexes (Murillo et al., 2019). Comparisons of isolation methods and the EVPsort resource further show that recovered RNA profiles depend on carrier enrichment and analytical workflow (Srinivasan et al., 2019; Chen et al., 2024). Current cancer rRF studies indicate that extracellular rRF profiles are influenced by carrier type and analytical workflow. Most studies have focused on extracellular abundance, biomarker performance, or intracellular effects after transfection, whereas the processes linking rRF release, cellular uptake, and downstream responses remain less well characterized. Clarifying these steps will help define the role of rRFs in intercellular communication (Yang et al., 2025; Gu et al., 2025; Zhan et al., 2025; Tosar et al., 2020; Murillo et al., 2019; Srinivasan et al., 2019; Chen et al., 2024).

### 4.3 Source ambiguity in cancer samples

The gastric study analyzed EV-enriched plasma RNA, the colorectal study analyzed plasma, and the pan-cancer study analyzed serum (Yang et al., 2025; Gu et al., 2025; Zhan et al., 2025). These materials are not interchangeable. Blood-cell composition, platelet activation, hemolysis, collection delay, and RNA isolation can influence the measured signal. The lung-cancer TRY-RNA study analyzed peripheral blood mononuclear cells, rather than a cell-free compartment, and combined tRNA-, rRNA-, and YRNA-derived species (Gu et al., 2020).

Microbial RNA creates another attribution problem. Ju et al. studied modifications of microbiome-derived cell-free RNA in colorectal-cancer samples (Ju et al., 2026). Microbiome-derived RNA should be distinguished from human rRFs when interpreting sequence origin, as this distinction is important for understanding the biological source and significance of extracellular RNA signals.

## 5 Biomarker applications and clinical interpretation

### 5.1 Tissue abundance and prognosis

In five paired hypopharyngeal tumors and adjacent tissues, PANDORA-seq showed a lower overall rsRNA fraction in tumors, with increased representation of 28S-derived and reduced representation of 18S-derived reads (Pu et al., 2025). These findings suggest a remodeling of the rsRNA composition in tumor tissue, characterized by shifts in the relative representation of 28S- and 18S-derived fragments rather than a uniform change across all rRFs.

A prostate tissue study compared 89 tumors with 53 benign prostatic-hyperplasia samples (Diamantop et al., 2024). The assayed 28S-rRF was lower in cancer overall, yet higher levels within the cancer group were associated with adverse pathological features and shorter biochemical-relapse-free survival after internal validation. Between-group diagnosis and within-group prognosis address different contrasts, so these directions are not necessarily contradictory. This candidate also cannot be equated with the rsRNA-28S–PTGIS fragment without sequence-level confirmation.

### 5.2 Circulating signatures and fragment chemistry

In gastric cancer, a plasma EV-enriched signature comprising the rRFs S2 and S7 and the tRF S10 yielded an AUC of 0.91 in 143 cases and 167 controls (Yang et al., 2025). Two independent test cohorts, including 47 cases versus 47 controls and 40 cases versus 43 controls, achieved AUCs of 0.97 and 0.91, respectively. In 20 patients, the levels of these RNAs decreased after surgery. In colorectal cancer, a six-rsRNA plasma panel achieved an AUC of 0.898, which increased to 0.942 when combined with conventional tumor markers (Gu et al., 2025). A pan-cancer study evaluated a six-exRNA classifier containing five rsRNAs in 120 serum samples (Zhan et al., 2025). The held-out subset was derived from the same overall cohort and therefore represented internal testing. Targeted nanopore sequencing further showed that the 28S4014–4035 fragment existed in four distinct 2′-O-methylation states in 43 individuals investigated for suspected lung cancer, including 27 cancer cases and 16 non-cancer controls (Sanchez-Delgado et al., 2025). The doubly methylated form was enriched in cancer cases, with an AUC of 0.84. Together, these studies highlight the potential of rRF abundance and chemical modification patterns as complementary biomarkers for cancer detection and clinical assessment.

### 5.3 Validation for clinical use

These studies are difficult to compare directly because of differences in cohorts, sample types, and analytical methods. Future clinical validation should focus on standardized procedures and independent prospective cohorts, particularly for diagnostic, prognostic, and monitoring applications. Mechanistic studies should further clarify localization, target dependence, and functional effects.

## 6 Therapeutic opportunities and translational requirements

### 6.1 Antisense inhibition and the direction of effect

An ASO strategy would be rational for an rRF whose endogenous activity sustains tumor growth or drug resistance. The H1299 rRF3 depletion phenotype provides an initial reason to test selective inhibition, although that study used vector-based RNA interference and did not establish an ASO therapy (Chen et al., 2017). Before developing an inhibitor, the fragment’s contribution must be reproduced with independent reagents and restored by an appropriate rescue construct.

The rsRNA-28S–PTGIS findings illustrate why the direction of intervention matters. ASO inhibition increased taxane resistance in the tested prostate-cancer cells (Qiao et al., 2023). These findings suggest that rsRNA-28S may have a protective role in taxane response and provide a rationale for exploring replacement strategies or downstream pathway modulation. The corresponding therapeutic strategies and key considerations are summarized in Table 2.

**TABLE 2:** Therapeutic strategies and the evidence needed to evaluate them.

Strategy	Existing rRF evidence	Proposed use	Required evidence
Inhibit a tumor-promoting fragment	rRF3 depletion alters H1299 phenotypes (Chen et al., 2017); this was vector-based RNA interference.	Evaluate a fragment-selective ASO if tumor dependence is confirmed.	Independent reagents; mature-fragment rescue; parental-rRNA selectivity; animal efficacy and safety.
Restore a protective fragment	rsRNA-28S ASO increases taxane resistance (Qiao et al., 2023).	Test a defined rsRNA-28S mimic as a resistance-modifying candidate.	Gain-of-function reversal, PTGIS-dependent rescue, dose and localization, combination efficacy *in vivo*.
Deliver S2 or S7 mimics	Reporter and protein responses after transfection in gastric-cancer cells (Yang et al., 2025).	Use as mechanistic probes; therapeutic direction is not established.	Endogenous necessity, tumor phenotype, target rescue, and effects on normal cells.
Engineer chemistry and delivery	Localization varies by sequence (Cherlin et al., 2024); terminal modification can alter innate sensing (Alharbi et al., 2026).	Test formulations that reach the required compartment.	Productive intracellular delivery, biodistribution, target engagement, immune effects, and tolerability.

These are candidate development strategies, not demonstrated clinical rRF therapies; ASO, antisense oligonucleotide; PTGIS, prostaglandin I2 synthase.

Selectivity is a central challenge because an rRF shares sequence with abundant parental rRNA and often with related fragments. An ASO may bind accessible parental RNA or several end variants. Candidate-directed studies should therefore measure parental rRNA integrity, ribosome function, and closely related fragments alongside the intended target. End-dependent recognition or differences in accessibility may offer selectivity, but must be demonstrated rather than assumed.

### 6.2 Synthetic mimics and replacement strategies

Synthetic rRF mimics offer a controllable approach for exploring replacement strategies, as their sequence, termini, and chemical modifications can be defined experimentally. In gastric-cancer cells, S2 and S7 mimics altered target-reporter activity and corresponding protein expression (Yang et al., 2025). These findings support the feasibility of using synthetic rRFs to modulate specific molecular pathways, although their therapeutic potential will depend on the biological role of the targeted pathway.

### 6.3 Delivery and preclinical evaluation

Candidate formulations could investigate local delivery, lipid-based systems, or engineered extracellular carriers, but detection of an rRF in EV-enriched plasma does not validate an EV delivery platform (Yang et al., 2025; Tosar et al., 2020; Murillo et al., 2019; Srinivasan et al., 2019; Chen et al., 2024). Uptake must be distinguished from productive delivery to the compartment where the fragment acts. Stability-enhancing chemical changes should be evaluated for their effects on target pairing, effector loading, and innate immune signaling; modification-dependent TLR7/8 effects provide a specific reason for this testing (Alharbi et al., 2026).

Preclinical development should establish exposure, biodistribution, target engagement, and dose-dependent efficacy in relevant tumor models, including drug combinations when resistance is the intended indication. Tests in normal cells and immune-competent settings should assess effects on ribosome function, inflammation, and systemic tolerability. The reviewed cancer rRF studies do not yet establish a clinically effective rRF-directed treatment. Their main therapeutic contribution is to nominate candidates and the experiments needed to evaluate them.

## 7 Limitations and unresolved questions

### 7.1 Inconsistent and negative observations

The literature does not support a uniform increase, decrease, or biological role for rRFs in cancer. Hypopharyngeal tumors had a lower overall rsRNA fraction, whereas selected circulating panels in other cancers were increased (Yang et al., 2025; Gu et al., 2025; Pu et al., 2025; Zhan et al., 2025). These observations concern different candidates and compartments and should not be collapsed into a common direction of regulation. Similarly, the adverse prognostic association of higher tissue 28S-rRF within prostate cancers does not invalidate its lower abundance relative to benign tissue, or establish identity with another 28S-derived candidate (Diamantop et al., 2024; Qiao et al., 2023).

Negative findings also constrain proposed mechanisms. Chen et al. did not detect double-stranded rRFs in their tick dataset, despite prior reports in other systems (Chen et al., 2017). This does not exclude rRF–mRNA duplexes, but it argues against assuming a universal double-stranded biogenesis intermediate. The nonsignificant chemotherapy-associated patient comparison and the lack of a proliferative effect after PTGIS overexpression likewise limit extrapolation from the prostate model (Qiao et al., 2023). Failure to demonstrate an endpoint must be distinguished from evidence that the endpoint is absent.

### 7.2 Experimental and cohort limitations

Mechanistic evidence is concentrated in a few cell systems. The H1299 and DU-145/DU-145R experiments do not establish activity across tumor lineages, and the gastric mimic experiments do not establish endogenous necessity. *In vivo* efficacy, physiological-dose rescue, and selective perturbation of a mature fragment remain insufficiently tested (Yang et al., 2025; Chen et al., 2017; Qiao et al., 2023). Cancer-associated expression, Ago recovery, target prediction, and reporter response therefore represent different stages of evidence rather than interchangeable proof of mechanism.

Clinical limitations include the five-pair hypopharyngeal study, the 23-patient prostate treatment comparison, and the 43-person lung methylation cohort (Pu et al., 2025; Sanchez-Delgado et al., 2025; Qiao et al., 2023). Although the gastric study included independent test cohorts, broader prospective and multicenter replication remains necessary (Yang et al., 2025). Fragment identity, carrier recovery, and analytical chemistry also vary across studies. These sources of heterogeneity preclude a pooled claim about all rRFs or direct comparison of published diagnostic AUCs.

### 7.3 Selection and publication bias

This narrative review has an incomplete historical search record and no quantitative assessment of publication bias. Candidate studies may preferentially report positive reporters, striking expression changes, or high-performing panels, while null perturbations, failed replications, and unsuccessful classifiers remain less visible. The extent of this bias is unknown. Preregistered analyses, transparent reporting of all tested candidates, sequence-level data sharing, and independent replication would make both positive and negative results more informative.

## 8 Conclusion

Cancer-associated rRFs comprise multiple molecular species with distinct origins, chemistries, and cellular contexts. The strongest current conclusions are candidate-specific: a limited set of perturbation studies supports cellular effects or target regulation, while a broader profiling literature supports exploratory diagnostic and prognostic associations. Non-canonical processing and extracellular fragmentation expand the possible origins of these signals but do not establish a shared cancer mechanism.

The next steps are to connect exact fragment identity to endogenous localization, selective perturbation, target dependence, and *in vivo* outcomes. Biomarker programs need standardized assays and prospective validation, while therapeutic programs must establish whether inhibition or replacement is appropriate for each candidate. Maintaining these distinctions will allow rRF research to develop clinically useful tests and testable therapeutic strategies without overstating the available evidence.
